# In Transgenic Erythropoietin Deficient Mice, an Increase in Respiratory Response to Hypercapnia Parallels Abnormal Distribution of CO_2_/H^+^-Activated Cells in the Medulla Oblongata

**DOI:** 10.3389/fphys.2022.850418

**Published:** 2022-04-19

**Authors:** Florine Jeton, Anne-Sophie Perrin-Terrin, Celine-Hivda Yegen, Dominique Marchant, Jean-Paul Richalet, Aurélien Pichon, Emilie Boncoeur, Laurence Bodineau, Nicolas Voituron

**Affiliations:** ^1^ Laboratoire “Hypoxie et Poumons”, UMR INSERM U1272, Université Paris 13, UFR SMBH, Bobigny, France; ^2^ Laboratory of Excellence (Labex) GR-Ex, PRES Sorbonne Paris Cité, Paris, France; ^3^ Inserm, UMR_S1158 Neurophysiologie Respiratoire Expérimentale et Clinique, Sorbonne Université, Paris, France

**Keywords:** erythropoietin, central CO_2_/H^+^ -activated cells, hypercapnia, retrotrapezoid nucleus/parafacial respiratory group, serotoninergic cells

## Abstract

Erythropoietin (Epo) and its receptor are expressed in central respiratory areas. We hypothesized that chronic Epo deficiency alters functioning of central respiratory areas and thus the respiratory adaptation to hypercapnia. The hypercapnic ventilatory response (HcVR) was evaluated by whole body plethysmography in wild type (WT) and Epo deficient (Epo-TAg^h^) adult male mice under 4%CO_2_. Epo-TAg^h^ mice showed a larger HcVR than WT mice because of an increase in both respiratory frequency and tidal volume, whereas WT mice only increased their tidal volume. A functional histological approach revealed changes in CO_2_/H^+^-activated cells between Epo-TAg^h^ and WT mice. First, Epo-TAg^h^ mice showed a smaller increase under hypercapnia in c-FOS-positive number of cells in the retrotrapezoid nucleus/parafacial respiratory group than WT, and this, independently of changes in the number of *PHOX2B*-expressing cells. Second, we did not observe in Epo-TAg^h^ mice the hypercapnic increase in c-FOS-positive number of cells in the nucleus of the solitary tract present in WT mice. Finally, whereas hypercapnia did not induce an increase in the c-FOS-positive number of cells in medullary raphe nuclei in WT mice, chronic Epo deficiency leads to *raphe pallidus* and *magnus* nuclei activation by hyperacpnia, with a significant part of c-FOS positive cells displaying an immunoreactivity for serotonin in the *raphe pallidus* nucleus. All of these results suggest that chronic Epo-deficiency affects both the pattern of ventilatory response to hypercapnia and associated medullary respiratory network at adult stage with an increase in the sensitivity of 5-HT and non-5-HT neurons of the raphe medullary nuclei leading to stimulation of *f*_R_ for moderate level of CO_2_.

## 1 Introduction

The glycoprotein erythropoietin (Epo) is known to play a key role in erythropoiesis, and insufficient Epo production in adults induces well-known blood defects, mostly due to the damage of Epo-producing cells or suppression of Epo production by inflammatory cytokines (Bunn, 2013). It has also been established that Epo has other functions, as evidenced by the fact that Epo and its receptor EpoR have been detected in many tissues (Rabie and Marti, 2008; Pichon et al., 2016). Notably, Epo and EpoR have been reported to be efficient at the level of the central nervous system in both normal brain development and in a protective effect in pathologies such as multiple sclerosis or epilepsy (Yu et al., 2002; Sättler et al., 2004; Chen et al., 2007; Kondo et al., 2009). Indeed, it has been reported that Epo deficiency produces deep alterations in the normoxic expression of hypoxia-related genes in the brain (El Hasnaoui-Saadani et al., 2009; Pichon et al., 2016). Thus, in normoxia, Epo-TAgh mice displayed an increase in transcript and protein levels of hypoxia inducible factors, vascular endothelial growth factor, erythropoietin receptors (EpoR), and neuronal NOS (El Hasnaoui-Saadani et al., 2009).

Several studies have documented the influence of Epo on the control of breathing. Brain Epo has been shown to modulate both the basal ventilation and hypoxic ventilatory response (Macarlupú et al., 2006; Soliz et al., 2007; Yalcin et al., 2007; Khemiri et al., 2012; Voituron et al., 2014; Ballot et al., 2015a; Caravagna et al., 2015; Caravagna and Soliz, 2015; Pichon et al., 2016). These observations can be related to the presence of EpoR in the main peripheral and central respiratory areas that regulate breathing (Soliz et al., 2005). On the basis of these data, and keeping in mind that the CO_2_/H^+^ regulation of breathing mainly depends on brainstem structures (Feldman et al., 2003; Guyenet et al., 2005, 2019; Nattie and Li, 2006a; Guyenet and Bayliss, 2015; Olivares et al., 2021), recent studies have aimed to determine if Epo modulates the hypercapnic ventilatory response to CO_2_ (HcVR). Searching for an influence of Epo in HcVR is relevant because the central CO_2_/H^+^ chemosensitivity is essential. Indeed, absence or drastic reduction of the CO_2_/H^+^ sensitivity leads to central hypoventilation syndromes such as Ondine’s curse (Weese-Mayer et al., 2010; Ramanantsoa et al., 2011). Conclusions of the few studies conducted on this topic do not reach consensus (Ballot et al., 2015b; Khemiri et al., 2016; Laouafa et al., 2016, 2017; Menuet et al., 2016; Silva et al., 2017). Menuet and collaborators reported a decrease in HcVR in adult transgenic mice over-expressing brain Epo, suggesting that an excess of Epo limits the CO_2_/H^+^ respiratory response (Menuet et al., 2016). This observation is in agreement with a study that used an exogenous supply of Epo in the brainstem of juvenile rats (Silva et al., 2017), but it is contradicted by others (Ballot et al., 2015b; Laouafa et al., 2016, 2017). In particular, transgenic newborn mice showing either a chronic deficient-expression of brain EPO or a pharmacologically-induced decrease in brain Epo do not show an increase in HcVR (Ballot et al., 2015b; Laouafa et al., 2017). We hypothesized that chronic life-long Epo deficiency is necessary to induce changes in the central CO_2_/H^+^-activated cells, and thus to observe an enhancement of the HcVR in accordance with the conclusion of Menuet and collaborators (Menuet et al., 2016). Changes in central CO_2_/H^+^-activated cells induced by chronic Epo deficiency are supported by the recognized link between neuronal plasticity phenomenon and over- or deficient-expression of Epo (Kamal et al., 2011; Voituron et al., 2014; Almaguer-Melian et al., 2015; Kimáková et al., 2017). Considering all these data, the present study investigated changes in the central CO_2_/H^+^-activated cells in chronic Epo deficient (Epo-TAg^h^) adult mice in parallel with the analysis of their HcVR. Because the major CO_2_/H^+^-chemosensitive sites, retrotrapezoid nucleus/parafacial respiratory group (RTN/pFRG) and medullary raphe serotoninergic neurons are located within the *medulla oblongata* (Mulkey et al., 2004; Hodges et al., 2008; Guyenet et al., 2013, 2019; Teran et al., 2014; Kumar et al., 2015; Ruffault et al., 2015), we decided to focus our investigations at the medullary level.

## 2 Materials and Methods

### 2.1 Ethical Approval

Experimental protocols were approved by the Ethics Committee for Animal Experiment Charles Darwin and the French Ministry of Research (Ce5/2011/05 and APAFIS#8192), done in accordance with the European Communities Council Directive of 22 September 2010 (2010/63/EU) for animal care, and conducted in accordance with the French legislation for animal care.

### 2.2 Animals and Procedures

All experiments were performed on in-house bread wild type (WT, *n* = 47) and Epo-TAg^h^ (*n* = 45) male adult littermates (≈10 weeks) from Bl6/CBA strain. The transgenic construct contains an SV40 sequence in the 5′ untranslated region of the mouse *Epo* gene, which is flanked on each side by 9 and 7.5 kb of DNA from the mouse *Epo* locus (Maxwell et al., 1993; Pichon et al., 2016). The *Epo* mutation reduces the whole body *Epo* expression (Binley et al., 2002) leading to plasma and brain Epo concentrations at approximately 50 pg/ml and 0.10 pg/mg respectively (around 150 pg/ml and 0.40 pg/mg for plasma and brain Epo concentration respectively in WT) (El Hasnaoui-Saadani et al., 2009). Thus, Epo-TAg^h^ and WT mice were selected on the basis of their genotype. The genomic DNA samples were prepared from the tail biopsies and PCR genotyping was performed using mouse epo-specific primers (mEpo forward: 5′-CGC​ACA​CAC​AGC​TTC​ACC​C-3′ and mEpo reverse: 5-CTG​TAG​GGC​CAG​ATC​ACC-3) and SV-40T antigen-specific reverse primer (5′-GCC​TAG​GCC​TCC​AAA​AAA​GC-3′). Mean body weight and temperature were 30.4 ± 3.3 g/35.3 ± 1.6°C for WT mice and 29.7 ± 4.0 g/35.0 ± 1.0°C for Epo-TAg^h^ mice respectively. Haemoglobin concentration and haematocrit were approximately 7 g/dl and 16–20% in Epo-TAg^h^ mice (approximately 17 g/dl and 38–40% in WT) (El Hasnaoui-Saadani et al., 2013). All animals were housed in a 12 h/12 h light/dark cycles at 18–20°C temperature and had *ad libitum* access to water and food.

### 2.3 Hypercapnic Ventilatory Response

#### 2.3.1 Recording of Ventilatory Variables

In non-anesthetized and unrestrained mice, ventilatory variables were recorded by whole-body plethysmography (WT *n* = 25; Epo-TAg^h^ *n* = 25) (Bartlett and Tenney, 1970; Voituron et al., 2014). The animal chamber (200 ml) was connected to a differential pressure transducer (model DP 45-18, Validyne Engineering Northridge, CA, United States), which measured pressure fluctuations within the chamber, relative to a reference chamber of the same volume. The differential pressure transduced signals were recorded by a Spike 2 data analysis system (CED, Cambridge United Kingdom). To avoid stress effects on ventilatory variables, mice were habituated in the recording chamber two or 3 days before the experiments. To evaluate the acute ventilatory response to hypercapnia, air was enriched with CO_2_ gas for 5 min (CO_2_ 4%, O_2_ 21%, balanced N_2_). At the beginning, mice were exposed to normoxic conditions for at least 30 min. Recordings were made under normoxic conditions for the last 10 min to define control values and then during the last min of hypercapnia. Only periods of breathing without body movements were analysed. We evaluated respiratory frequency (*f*_R_, cycles. min^−1^), tidal volume (V_T_, µl) normalized as the ratio of V_T_ divided by body weight (V_T_, µl.g^−1^) and minute ventilation (V̇_E_, ml.g^−1^. min^−1^). For V_T_ calculation, determined using Drorbaugh and Fenn (1955), the rectal temperature was measured just before placing mice in the recording chamber for normoxic values and just after the end of CO_2_ exposure for hypercapnic values.

#### 2.3.2 Analyses of Ventilatory Variables

Values are presented as mean ± standard deviation (SD). The Kolmogorov–Smirnov test assessed the normality of distribution. The effects of hypercapnia on ventilatory variables in male WT and Epo-TAg^h^ mice were evaluated by multivariate analyses of variance (MANOVA) with Greenhouse and Geisser adjustments. All analyses were performed with the Statistica software (Stat Soft, Tulsa, United States). Differences were considered significant when *p* < 0.05.

### 2.4 Analysis of Hypercapnia-Responding Areas of the Medulla Oblongata

#### 2.4.1 Immunohistological Procedures

To localize medullary areas presenting modifications of activity in response to hypercapnia, we analysed *c-fos* expression in WT (*n* = 22) and Epo-TAg^h^ (*n* = 20) mice. The c-FOS immunodetection is a classic tool used to identify central pathways involved in specific physiological response that requires minimizing manipulations that could induce changes of cell activity unrelated to the studied stimulus and a sufficiently long induction period to induce detectable changes in *c-fos* expression (Larnicol et al., 1994; Herdegen and Leah, 1998; Bodineau and Larnicol, 2001; Okada et al., 2002; Perrin-Terrin et al., 2016). Animals were placed in an airtight box ventilated with hypercapnic gas mixture (CO_2_ 4%, O_2_ 21% balanced N_2_; WT, *n* = 12 and Epo-TAg^h^, *n* = 10) or normocapnic gas mixture (O_2_ 21% balanced N_2_; WT, *n* = 10 and Epo-TAg^h^, *n* = 10) for 2 h. At the end of the hypercapnic or normocapnic period, mice were deeply anesthetized (pentobarbital, 100 mg/kg, i.p.) and transcardially perfused with saline solution (NaCl 0.9%), and then with 4% paraformaldehyde in 0.1 M phosphate buffer (pH7.4). Brains were removed, postfixed in the same fixative solution for 48 h and then cryoprotected in 30% sucrose in 0.1 M PBS for 24–48 h. Standard immunohistochemical procedures were used to locate c-FOS on 40 μm-thick coronal free-floating sections of the medulla oblongata obtained using a cryostat (Leica CM1510S or Leica CM1850UV).

The detection of c-FOS was coupled with that of serotonin (5-hydroxytryptamine, 5-HT) or Glial fibrillary acidic protein (GFAP) to characterize the cells displaying changes in activity revealed by c-FOS analysis. The detection of PHOX2B was also used to search for changes between in Epo-TAg^h^ and WT mice at the level of the main CO_2_/H^+^-sensor of the *medulla oblongata*, the retrotrapezoid nucleus/parafacial respiratory group (RTN/pFRG) (Mulkey et al., 2004; Guyenet et al., 2013; Kumar et al., 2015; Ruffault et al., 2015).

The manufacturer verified the specificity of primary antibodies in all cases and in addition, control sections were processed in parallel, but with the omission of the primary or secondary antibodies; we observed no labeling in the absence of the primary or secondary antibodies.

#### 2.4.2 c-FOS Immunohistochemistry

Sections were placed in 0.1 M phosphate-buffer saline solutions supplemented with 0.3% Triton X-100 and 2% normal goat serum, and then incubated for 48 h at 4°C with a rabbit polyclonal antibody against c-FOS (sc-52; Santa Cruz Biotechnology Inc., Santa Cruz, CA, United States), diluted 1/2000 in 0.1 M phosphate-buffer saline solution supplemented with 0.3% Triton X-100 and 0.25% bovine serum albumin. Sections were then incubated for 1 h with a biotinylated goat anti-rabbit immunoglobulin diluted 1/500, and for 1 h with an avidin-biotin complex (ABC, Novostain Super ABC Kit, Novocastra Laboratories, Newcastle, United Kingdom). Peroxidase activity was detected by using VECTOR NovaRED (Substrate Kit for Peroxidase, Vector Laboratories, Burlingame, CA, United States). Sections were mounted in sequential caudo-rostral order on slides, air-dried, dehydrated with absolute alcohol, cleared with xylene and coverslipped with mounting medium (Entellan^®^, VWR International S.A.S).

#### 2.4.3 5HT and c-FOS Double Labelling

5-HT cells of medullary raphe nuclei are considered as important CO_2_/H^+^-chemosensitive sites (Hodges et al., 2008; Teran et al., 2014). In order to determine the serotoninergic phenotype of hypercapnic activated-cells in medullary midline raphe, both c-FOS and 5HT were investigated. Sections were first incubated with a polyclonal rabbit anti-c-FOS primary antibody (sc-253; Santa Cruz Biotechnology Inc., United States; 1:8,000; 48 h at 4°C), then with biotinylated goat anti-rabbit immunoglobulin (BA-1000; Vector Laboratories, Canada; 1:500) in 1% normal goat serum supplemented with 0.3% Triton X-100 for 2 h. After 1 h with ABC, peroxidase activity was detected with 0.02% 3,3′-diaminobenzidine tetrahydrochloride, 0.04% nickel ammonium sulphate and 0.01% hydrogen peroxide in Tris-HCl buffer (pH 7.6). Subsequently, sections were incubated with blocking serum (1% bovine serum albumin) for 1 h and then with a polyclonal rabbit anti-5-HT primary antibody (sc-73024; Santa Cruz Biotechnology Inc., United States; 1:500, 48 h at 4°C). Then sections were incubated with biotinylated goat anti-rabbit immunoglobulin (BA-1000; Vector Laboratories, Canada; 1:500) in 1% bovine serum albumin for 2 h and then with ABC for 1 h. Peroxidase activity was detected by using VECTOR NovaRED. Sections were mounted in sequential caudo-rostral order on slides, air-dried, dehydrated with absolute alcohol, cleared with xylene and coverslipped with mounting medium (Entellan^®^, VWR International S.A.S).

#### 2.4.4 GFAP and c-FOS Double Labelling

Astrocytes, a well-known source of Epo, are involved in the modulation of neighbouring neurons at the RTN/pFRG place (Marti et al., 1996; Gourine et al., 2010; Olivares et al., 2021). To determine if the RTN/pFRG hypercapnic c-FOS-positive cells were astrocytes, double labelling c-FOS and GFAP was performed. Sections were first incubated with a polyclonal rabbit anti-c-FOS primary antibody (sc-253; Santa Cruz Biotechnology Inc., United States; 1:2000; 48 h at 4°C), then with biotinylated goat anti-rabbit immunoglobulin (BA-1000; Vector Laboratories, Canada; 1:500) in 1% normal goat serum supplemented with 0.3% Triton X-100 for 1 h. After 1 h with ABC, peroxidase activity was detected with 0.015% 3,3′-diaminobenzidine tetrahydrochloride, 0.4% nickel ammonium sulfate and 0.01% hydrogen peroxide in Tris-HCl buffer (pH 7.6). Subsequently, sections were incubated with blocking serum (1% bovine serum albumin) for 1 h and then with a polyclonal rabbit anti-GFAP primary antibody (Z0334; Dako, Denmark; 1:1,000, 48 h at 4°C). After that, sections were incubated with biotinylated goat anti-rabbit immunoglobulin (BA-1000; Vector Laboratories, Canada; 1:500) in 1% bovine serum albumin for 1 h at room temperature and then with ABC for 1 h. Peroxidase activity was detected with 0.02% 3,3′-diaminobenzidine tetrahydrochloride and 0.01% hydrogen peroxide in Tris-HCl buffer (pH 7.6). Sections were mounted in sequential caudo-rostral order on slides, air-dried, dehydrated with absolute alcohol, cleared with xylene and coverslipped with mounting medium (Entellan^®^, VWR International S.A.S).

#### 2.4.5 PHOX2B Immunohistochemistry

CO_2_/H^+^-activated cells of the RTN/pFRG are described as PHOX2B-positive (Mulkey et al., 2004; Guyenet et al., 2013; Kumar et al., 2015; Ruffault et al., 2015). To compare distribution of PHOX2B-positive neurons in RTN/pFRG between WT and Epo-TAg^h^ mice, sections were incubated with a polyclonal goat anti-PHOX2B antibody (sc-13226; Santa Cruz Biotechnology Inc., United States; 1:750) in 1% bovine serum albumin for 48 h at 4°C. Afterward, sections were firstly incubated with a biotinylated horse anti-goat immunoglobulin (BA-9500; Vector Laboratories, Canada; 1:500) in 1% bovine serum albumin for 2 h, and then with ABC in 1% bovine serum albumin for 1 h, respectively. Peroxidase activity was detected with 0.02% 3,3′-diaminobenzidine tetrahydrochloride and 0.01% H_2_O_2_ in 0.005 M Tris-HCL buffer. Sections were mounted in sequential caudo-rostral order on slides, air-dried, dehydrated with absolute alcohol, cleared with xylene and coverslipped with mounting medium (Entellan^®^, VWR International S.A.S).

#### 2.4.6 Quantitative Analysis of the Effect of Hypercapnia on the Number of c-FOS-Positive Cells and Their Characterization

Sections were examined under a light microscope (Zeiss axioskop, Germany or Leica DM 2000; Leica Microsystems, Heidelberg, Germany) and regions of interest were photographed with a digital camera (Q-Imaging Retiga-200R CCD or Leica DFC450 C, Germany).

We analyzed c-FOS-positive cells in structures of the *medulla oblongata* related to the respiratory control and its chemical regulation at high magnification (×200) using standard landmarks (Paxinos et al., 2001). The number of c-FOS-positive cells was determined in the nucleus of the solitary tract (NTS), which includes three subdivisions: commissural (cNTS), medial (mNTS) and ventrolateral (vlNTS), the medullary *raphes* nuclei (*Raphe Osbcurus* nucleus, ROb; *Raphe Pallidus* nucleus, RPa and *Raphe Magnus* nucleus, RMg), the ventrolateral reticular nucleus of the medulla (VLM), the facial nucleus (n7), and the ventral medullary surface (VMS). Using standards landmarks (Paxinos et al., 2001; Voituron et al., 2011), the VMS was sub-divided in two areas corresponding to the retrotrapezoid nucleus/parafacial respiratory group (RTN/pFRG, under the facial nucleus) and the parapyramidal area (PP; lateral edge of the pyramidal tract) (Voituron et al., 2011). The VLM was separated in caudal and rostral parts, cVLM from the pyramidal decussation to the caudal edge of the lateral paragigantocellular nucleus and rVLM from the caudal edge of the lateral paragigantocellular nucleus to the caudal edge of the facial nucleus (Voituron et al., 2011). We localized all of these structures with the aid of numerous ventral, dorsal, and lateral landmarks to delimit the entire volume of each structure.

For each analysed area, the mean number of c-FOS-positive cells per section was calculated on one side for bilateral structures (mNTS, vlNTS, cVLM, rVLM, RTN/pFRG, PP, RMg and n7) and on the entire area for midline structures (cNTS, RPa, ROb). A Kolmogorov–Smirnov test assessed the normality of distribution. Differences between mean numbers of c-FOS-positive cells per structure obtained under control or hypercapnic conditions in WT and Epo-TAg^h^ mice were analysed by multivariate analyses of variance (MANOVA). Newman Keuls post-hoc tests were used to assess specific differences between groups. All analyses were performed with the Statistica software (Stat Soft, Tulsa, United States). Differences were considered significant when *p* < 0.05.

## 3 Results

### 3.1 Minute Ventilation in Normocapnia was not Modified in Epo-TAg^h^ Compared to WT Mice

**FIGURE 1:**
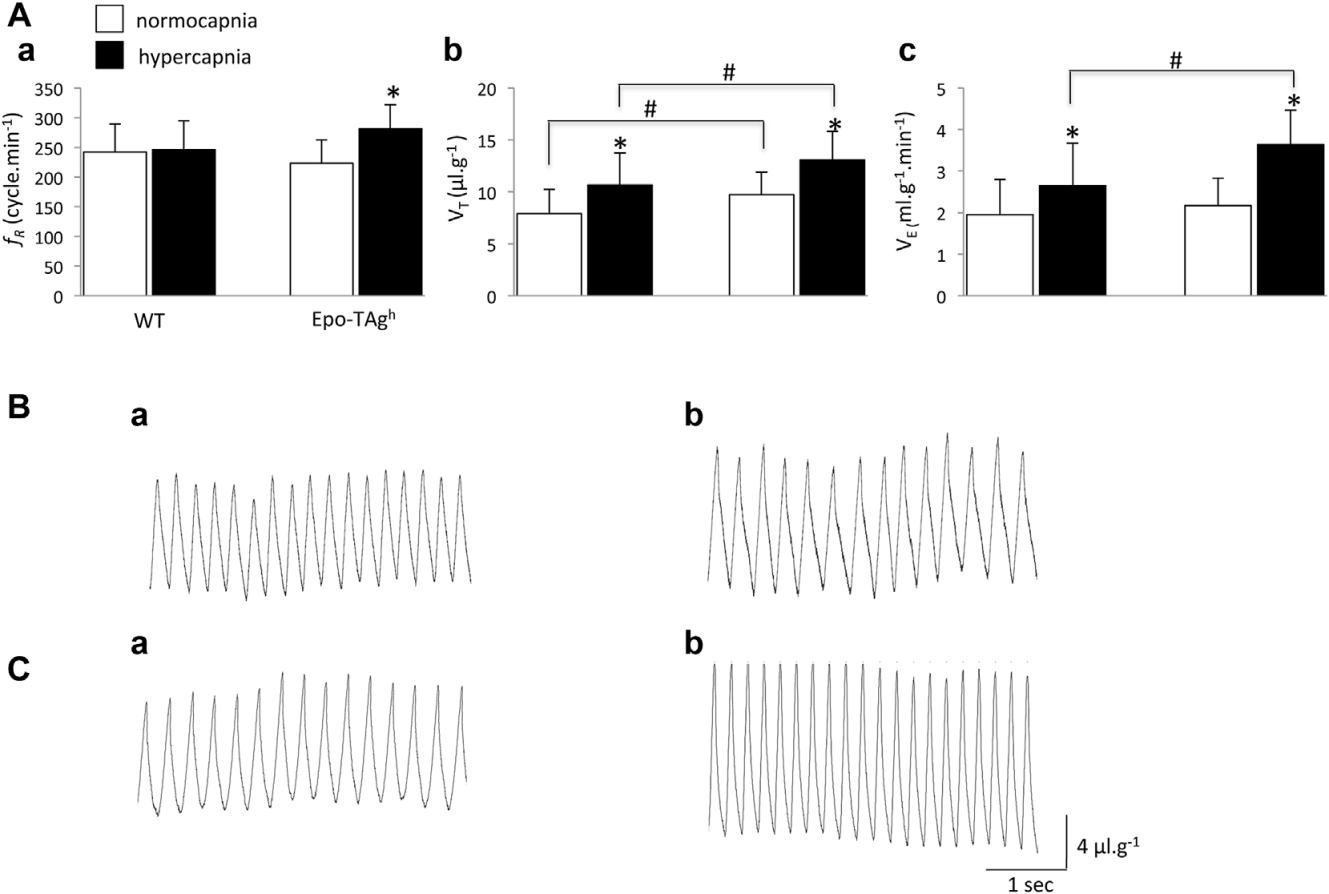
Chronic Epo-deficiency led to an increase in the ventilatory response to hypercapnia with change of ventilatory pattern compared to WT mice. Histograms showing mean values of respiratory variables of WT and Epo-TAg^h^ mice; respiratory frequency (*f*_R_, **(Aa)**), tidal volume (VT, **(Ab)**) and minute ventilation (V̇_E_, **(Ac)**) under normocapnia (white bars) and hypercapnia (black bars). Traces illustrate V̇_E_ of WT **(B)** and Epo-TAg^h^ **(C)** mice under normocapnia **(Ba,Ca)** and hypercapnia **(Bb,Cb)**. Data are expressed as mean ± SD. *indicates a significant difference between normocapnia *vs*. hypercapnia (same strain) and # indicates a significant difference between WT *vs*. Epo-TAg^h^ mice. * and #*p* < 0.05.

Although Epo-TAg^h^ mice displayed a significant increase in V_T_ compared to WT (∼23%; Figures 1Ab,Ba,Ca), V̇_E_ was not different between WT and Epo-TAg^h^ male mice in normocapnic condition (Figures 1Ac,Ba,Ca).

### 3.2 Ventilatory Pattern in Response to Hypercapnia Differed Between Epo-TAg^h^ and WT Mice

In WT mice, hypercapnia induced a significant increase in V_T_ (∼35%; Figures 1Ab, Bb) without change in *f*_R_ (Figure 1Aa) whereas in Epo-TAg^h^ mice hypercapnia significantly increased both V_T_ and *f*_R_ (∼34 and ∼26%, respectively; Figures 1Aa,Ab,Cb). It should be noted that in absolute values, the V_T_ in Epo-TAg^h^ mice was significantly larger than in WT mice (Figures 1Ab,Cb). As a result, the hypercapnic increase in V̇_E_ was significantly greater in Epo-TAg^h^ than in WT mice (∼68 and ∼36%, respectively; Figures 1Ac,Bb,Cb).

### 3.3 Medullary Areas Involved in the HcVR Were Different Between WT and Epo-Tag^h^ Mice

**FIGURE 2:**
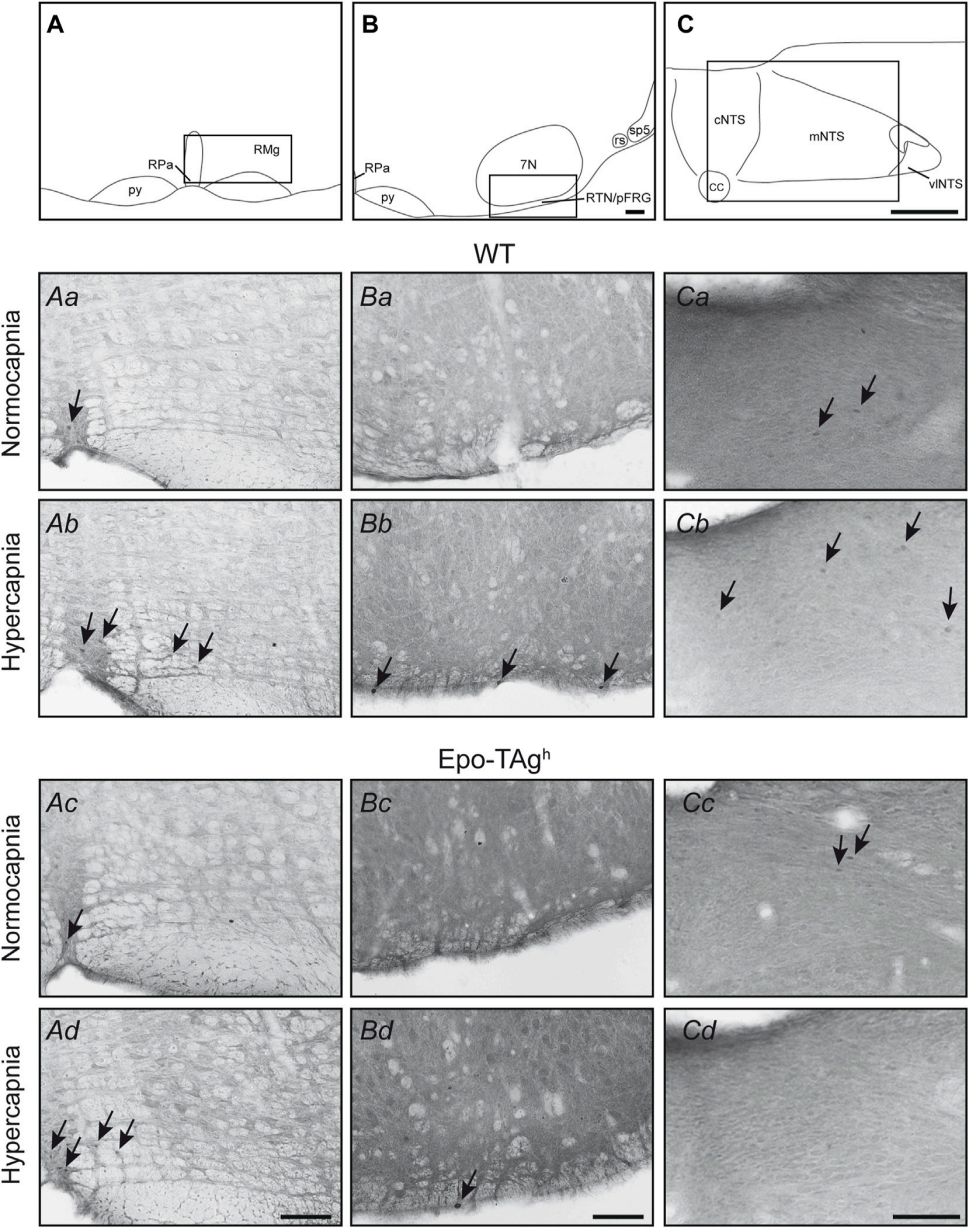
Epo deficiency affected the medullary respiratory network activated by hypercapnia. Drawings locating the structures illustrated in the microphotographs below, *raphe pallidus* and *magnus* nuclei **(A)**, retrotrapezoid nucleus/parafacial respiratory group (RTN/pFRG) **(B)**, and nucleus of the solitary tract **(C)**. The black box defines the photographed areas. Photomicrographs of sections immunolabelled for c-FOS in WT **(Aa,Ba,Ca,Ab,Bb,Cb)** and Epo-Tag^h^ **(Ac,Bc,Cc,Ad,Bd,Cd)** mice in *raphe magnus* and raphe *pallidus* nuclei **(Aa–Ad)** under normocapnia **(Aa,Ac)** and hypercapnia **(Ab,Ad)**, in retrotrapezoid nucleus/parafacial respiratory group **(Ba–Bd)** under normocapnia **(Ba,Bc)** and hypercapnia **(Bb,Bd)**, and in nucleus of the solitary tract **(Ca–Cd)** under normocapnia **(Ca,Cc)** and hypercapnia **(Cb,Bd)**. Black arrows show some c-FOS-positive neurons. Scale bars = 100 µm. Abbreviations: 7N, facial nucleus; cc: central canal; cNTS, commissural part of the nucleus of the solitary tract; mNTS, medial part of the nucleus of the solitary tract; py, pyramidal tract; RMg, *raphe magnus* nucleus; RPa, *raphe pallidus* nucleus; rs, rubrospinal tract; RTN/pFRG, retrotrapezoid nucleus/parafacial respiratory group; sp5, spinal trigeminal tract; vlNTS, ventrolateral part of the nucleus of the solitary tract.

In normocapnic conditions, we observed basal *c-fos* expression in most studied respiratory structures (Table 1). There was no difference in *c-fos* expression in baseline condition between WT and Epo-TAg^h^ mice (Table 1, Figures 2Aa,Ba,Ca,Ac,Bc,Cc).

**TABLE 1:** Average number of C-FOS-positive cells in respiratory areas of the medulla oblongata under normocapnia (Nc) and hypercapnia (Hc) in male wild type (WT) and Epo-TAg^h^ mice.

	WT		Epo-TAg^h^	
	Nc	Hc	Nc	Hc
cNTS	0.8 ± 1.0	1.7 ± 1.4*	0.2 ± 0.2	0.2 ± 0.2^$^
mNTS	0.7 ± 0.8	2.0 ± 1.0*	0.5 ± 0.4	0.5 ± 0.3^$^
vlNTS	0.2 ± 0.3	0.4 ± 0.3	0.2 ± 0.2	0.2 ± 0.2
cVLM	0.1 ± 0.1	0.4 ± 0.8	0.2 ± 0.4	0.5 ± 0.4
rVLM	0.4 ± 0.3	1.4 ± 0.9*	0.2 ± 0.3	0.7 ± 0.4^$^
RTN/pFRG	0.6 ± 0.3	2.2 ± 0.9*	0.3 ± 0.2	0.9 ± 0.4*^$^
PP	0.5 ± 0.3	1.0 ± 0.5	0.8 ± 0.5	1.1 ± 0.7
RPa	0.8 ± 0.5	2.4 ± 1.1	1.2 ± 1.3	3.6 ± 2.9*
RMg	0.2 ± 0.2	2.2 ± 1.8	0.2 ± 0.3	3.1 ± 3.5*
ROb	0.1 ± 0.1	0.1 ± 0.1	0.0 ± 0.0	0.0 ± 0.0
N7	0.0 ± 0.0	0.0 ± 0.0	0.1 ± 0.1	0.1 ± 0.1

Mean ± SD. VLM, ventrolateral reticular nucleus of the medulla; RTN/pFRG, retrotrapezoid nucleus/parafacial respiratory group; RPa, *raphe pallidus* nucleus; RMg, *raphe magnus* nucleus; ROb, *raphe obscurus* nucleus; PP, parapyramidal area; cNTS, commissural part of the nucleus tractus solitary; mNTS, medial part of the nucleus tractus solitary; vlNTS, ventrolateral part of the nucleus tractus solitary; N7, facial nucleus. Note that around 25 slices per mice were used to count c-Fos positive cells. *p < 0.05 Nc *vs*. Hc (same strain); $ *p* < 0.05 WT, *vs*. EpoTAg^h^.

The hypercapnic exposure led to an increase in *c-fos* expression in WT mice in cNTS (∼112%), mNTS (∼185%), rVLM (∼250%) and RTN/pFRG (∼267%) but not in other analysed medullary areas (Table 1, Figures 2Ab,Bb,Cb).

**FIGURE 3:**
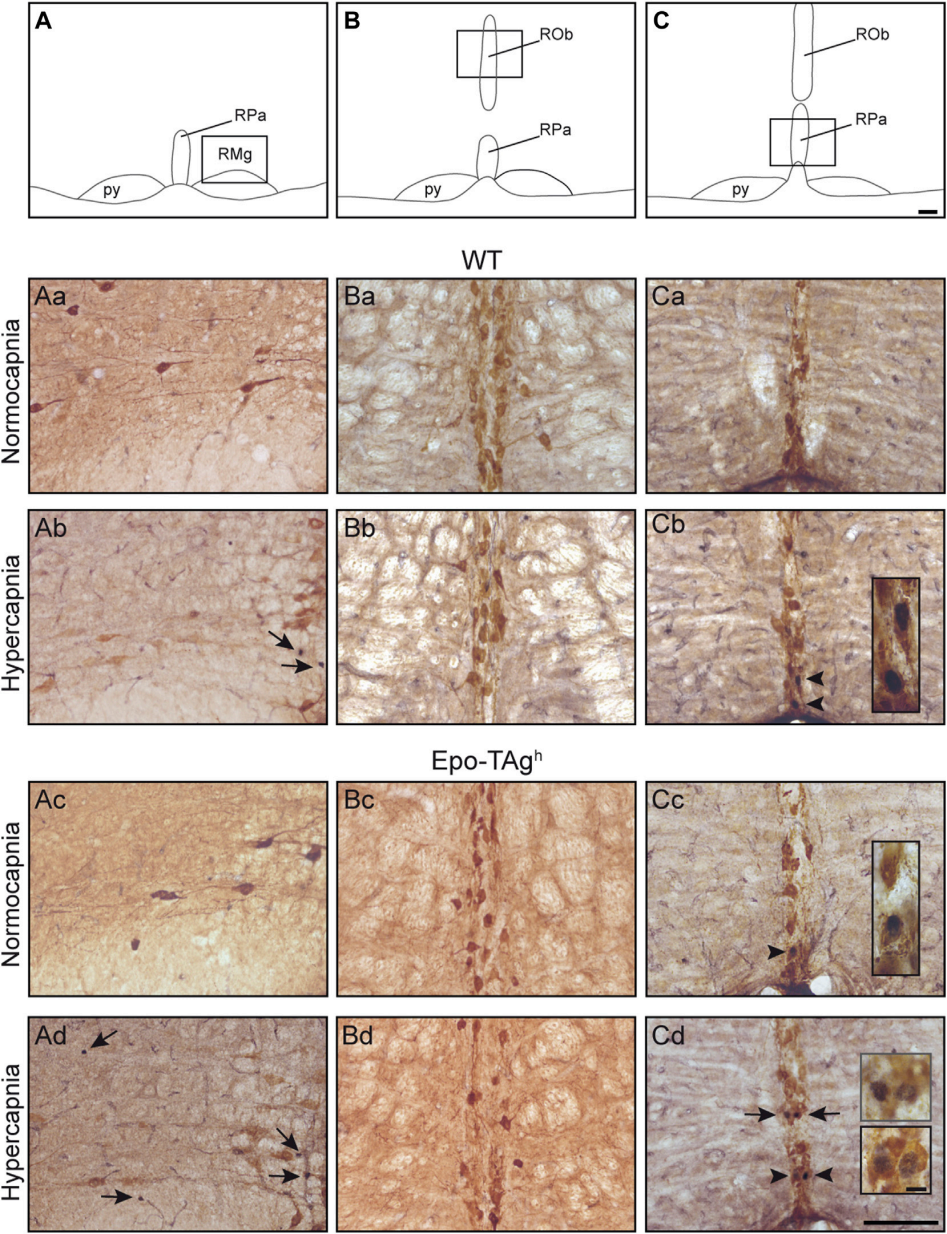
Serotoninergic character of hypercapnic c-FOS positive cells in medullary raphe nuclei. Drawings of the medulla oblongata locating 5-HT structures illustrated in the microphotographs below, *raphe magnus* **(A)**, *raphe obscurus* **(B)**, and *raphe pallidus* nucleus **(C)** nuclei. Scale bar = 100 μm. The black box defines the photographed areas. Photomicrographs of sections dually immunolabelled for c-FOS and 5-HT in WT **(Aa,Ba,Ca,Ab,Bc,Cb)** and Epo-Tag^h^ **(Ac,Bc,Cc,Ad,Bd,Cd)** mice in raphe *magnus* nucleus **(Aa–Ad)** under normocapnia **(Aa,Ac)** and hypercapnia **(Ab,Ad)**, in raphe *obscurus* nucleus **(Ba–Bd)** under normocapnia **(Ba,Bc)** and hypercapnia **(Bb,Bd)**, and in nucleus *pallidus* nucleus **(Ca–Cd)** under normocapnia **(Ca,Cc)** and hypercapnia **(Cb,Cd)**. Black arrows show c-FOS positive neurons and black arrowheads **(Cb,Cd)** show c-FOS/5-HT-positive neurons. Scale bars = 100 µm. High magnification in **(Cb–Cd)**, scale bar = 10 μm. Abbreviations: py, pyramidal tract; RMg, *raphe magnus* nucleus; ROb, *raphe obscurus* nucleus; RPa, *raphe pallidus* nucleus.

**FIGURE 4:**
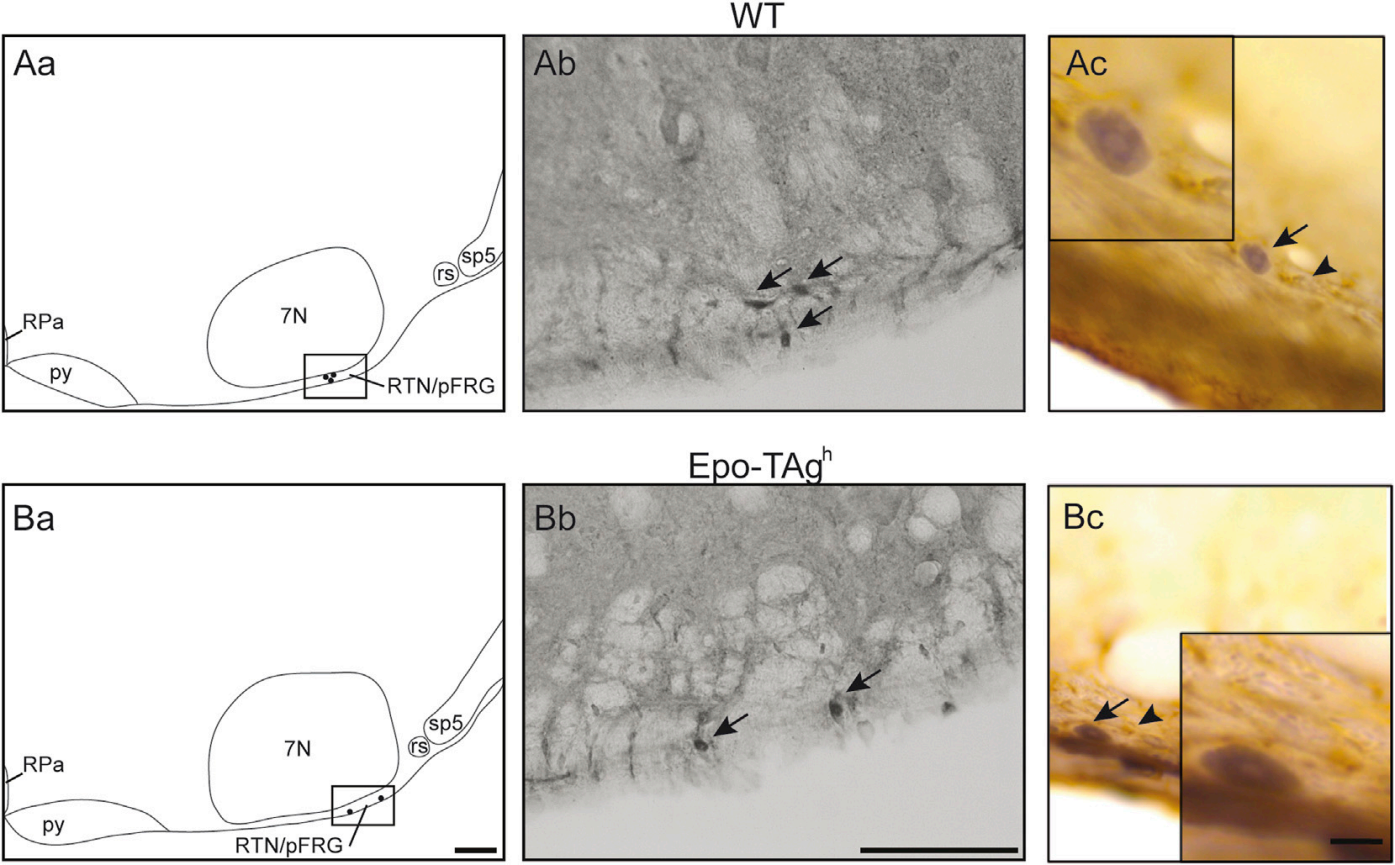
Epo deficiency did not modify *PHOX2B* expression in RTN/pFRG and C-FOS positive cells of the RTN/pFRG did not express GFAP in WT and Epo-TAg^h^ mice. Drawings of representative section from the ventral medulla oblongata illustrating the distribution of PHOX2B-positive cells (black points) in the retrotrapezoid nucleus/parafacial respiratory group of WT **(Aa)** and Epo-TAg^h^ **(Ba)** mice. Scale Bar = 100 µm. The black box defines the photographed area in the right of drawings. Photomicrographs illustrating the immunolabelling for PHOX2B in the RTN/pFRG of WT **(Ab)** and Epo-TAg^h^ **(Bb)** mice in normocapnia. Black arrows indicate PHOX2B-positive cells. Scale bar: 100 µm. Photomicrographs in the RTN/pFRG of a section dually immunolabelled for c-FOS and GFAP in WT **(Ac)** and Epo-TAg^h^ **(Bc)** mice in hypercapnia. Black arrows showed c-FOS positive neurons nuclei and black arrowheads showed GFAP-positive astrocytic extensions. Note that GFAP-cells were not c-FOS positive. The inserts show an enlargement of the area of interest. Scale bar = 10 µm. Abbreviations: 7N, facial nucleus; py, pyramidal tract; rs, rubrospinal tract; sp5, spinal trigeminal tract; RPa, *raphe pallidus* nucleus; RTN/pFRG, retrotrapezoid/parafacial respiratory group.

In Epo-TAg^h^ mice, c-FOS distribution was different from WT mice. No modification of *c-fos* expression was found in NTS and VLM (Table 1, Figure 2Cd). Epo-TAg^h^ mice displayed an hypercapnic increase in the number of c-FOS positive cells in RPa (∼200%) and RMg (∼1,450%; Table 1, Figure 2Ad). Although no co-labelled cells were observed in the RMg and ROb of both Epo-TAg^h^ and WT mice (Figures 3Ab,Ad,Bd), in the RPa, approximately 66% of c-FOS positive cells were also immunoreactive for 5-HT in WT mice in hypercapnic conditions (Figure 3Cb) and they were approximately 75% in Epo-TAg^h^ mice (Figure 3Cd). This observation mainly concerned the rostral part of the RPa (Figure 3C). In addition, we observed a significant increase in *c-fos* expression in RTN/pFRG (∼200%; Table 1, Figure 2Bd), but this increase was significantly reduced compared to that observed in WT mice (Table 1, Figure 2Bb). PHOX2B immunodetection in the RTN/pFRG suggested that this under-activation did not depend on a smaller number of PHOX2B-cells in this area in Epo-TAg^h^ mice compare to WT mice (Figure 4). In addition, the analysis of *c-fos* and *Gfap* expression in WT and Epo-TAg^h^ mice didn’t show any double-labelled cells in the RTN/pFRG (Figure 4).

## 4 Discussion

We described the CO_2_/H^+^-activated cells in the *medulla oblongata* in Epo deficient adult mice by analysing c-FOS protein levels by immunohistochemistry. Our main finding is that there were differences in the medullary CO_2_/H^+^-activated cells between Epo-TAg^h^ and their littermate WT mice at the level of major CO_2_/H^+^-chemosensitive sites *i.e.* RTN/pFRG region and medullary raphe nuclei. These changes are possibly related to neuronal plasticity, as already shown under chronic Epo over- or deficient-expression (Kamal et al., 2011; Voituron et al., 2014; Almaguer-Melian et al., 2015; Kimáková et al., 2017). The changes in CO_2_/H^+^-activated cells observed in Epo-TAg^h^ coincided with a change in the pattern and a greater response to CO_2_ as compared to WT mice.

### 4.1 Methodological Considerations

The impact of Epo in the neural control of breathing is sex-specific (Iturri et al., 2017; Jeton et al., 2017). In this work we investigated the impact of Epo-deficiency on hypercapnic ventilatory response in adult male mice in order to avoid the influence of female sexual hormone fluctuation on our results. In addition, we chose to study the ventilatory response to hypercapnia in adults in order to have a stable and robust response. Indeed, like many physiological regulations, the ventilatory response to hypercapnia matures with age (Bissonnette and Knopp, 2004; Putnam et al., 2005). Finally, our model combines the effects of chonic Epo deficiency and the effect of chronic anemia (Low O_2_ content) (Pichon et al., 2016). Thus, we cannot exclude that each affect neuronal plasticity.

c-FOS protein detection is classically used as a marker of neuronal activity (Dragunow and Faull, 1989) to highlight central pathway involved in specific physiological responses (Perrin-Terrin et al., 2016). This technique allows determination of cell populations that present modification of their activity in response to stimulus such as hypercapnia. However, interpretation of the results must take into account some limitations. Indeed, c-Fos expression depends on intensity of the stimulus, time of exposure, etc. (Morgan et al., 1987; Marina et al., 2002; Perrin-Terrin et al., 2016). In this study, we used a moderate stimulation (4% CO_2_ vs. 8–15% in other studies (Sato et al., 1992; Miura et al., 1994; Okada et al., 2002)), which may explain the low number of c-FOS positive neurons in the present study. Supporting this explanation, another study analysing changes in c-FOS-positive number of cells in respiratory areas in response to the same range of hypercapnia as us (CO_2_ 5%) obtained similar c-FOS quantitative results to those of the present work (Berquin et al., 2000). Moreover, some respiratory neurons may not express c-Fos even under strong excitatory respiratory stimulation (Song et al., 2012). Finally, a difference in the intensity of the hypercapnic stimulation could therefore be at the origin of the apparent discrepancy between our results and those in the literature. It should also be noted that the quantitative results in terms of number of c-FOS positive neurons that we obtained were compared between WT and Epo mice deficient in normocapnia and hypercapnia and were not directly compared with those obtained during previous studies.

### 4.2 Effect of Chronic Epo Deficiency on Ventilatory Response to Hypercapnia

Our results showed that the ventilatory response to hypercapnia in WT mice is mainly due to an increase in V_T_. This result is in agreement with previous studies, which suggested that CO_2_-homeostasis was regulated by an increase in V_T_ at the moderate level of CO_2_ used in the present study (Duffin et al., 2000; Ohashi et al., 2013). In contrast, Epo-TAg^h^ mice increased both V_T_ and *f*_R_ in response to 4% of CO_2_, which is classically observed in response to a more elevated level of CO_2_ (Duffin et al., 2000). These observations suggest that chronic Epo deficiency leads to changes in mechanisms at the origin of the ventilatory drive adaptation to hypercapnia, especially through an increase in sensitivity of the mechanisms that participate to the increase in *f*_R_. This hypothesis is supported by the distribution of c-FOS-positive cells under 4% CO_2_ hypercapnia, which differ between Epo-TAg^h^ mice and their littermates WT. The mechanisms by which Epo may affect the ventilatory response to hypercapnia are debated. Recent studies suggest that Epo overexpression in brain and/or circulation (Tg6 and Tg21 mice) blunts HcVR. However one study suggested that Epo, by interacting with central and peripheral chemoreceptors, blunts this ventilatory response (Menuet et al., 2016) while other study suggests that the decrease was more likely related to a reduction in the hypercapnic metabolism (Laouafa et al., 2016).

### 4.3 The Retrotrapezoid Nucleus/Parafacial Respiratory Group Displays Decreased c-Fos Expression Induced by Hypercapnia in Epo Deficient Mice

The increase in c-FOS positive cell number in RTN/pFRG under hypercapnia was lower in Epo-TAg^h^ mice than in WT. As RTN/pFRG is considered as a major CO_2_/H^+^-chemosensitive site (Mulkey et al., 2004; Guyenet et al., 2013, 2019; Kumar et al., 2015; Ruffault et al., 2015), this result suggested a smaller number of CO_2_/H^+^-sensitive neurons in this area or a decrease in their sensibility to CO_2_/H^+^. RTN/pFRG CO_2_/H^+^-sensitive neurons are PHOX2B-positive (Guyenet and Bayliss, 2015; Ruffault et al., 2015). We thus searched for a decrease in PHOX2B-positive number of cells in the RTN/pFRG in Epo-TAg^h^ compared to WT, and found that Epo-TAg^h^ mice have the same number of PHOX2B-positive neurons as compared to WT mice. It was therefore not probable that the decrease in hypercapnic *c-fos* expression in Epo-TAg^h^ mice depended on a loss of PHOX2B-CO_2_/H^+^-sensitive cells in this area, but rather to a reduction of their CO_2_/H^+^-sensitivity. CO_2_/H^+^-activation processes for RTN/pFRG neurons have been associated with direct activation of two molecular proton detectors, GPR4 and TASK-2 (Gestreau et al., 2010; Wang et al., 2013; Guyenet and Bayliss, 2015; Kumar et al., 2015). Indeed it has been suggested that Epo may target RTN neurons and increase the efflux of potassium through TASK-2 channels (Menuet et al., 2016). Therefore, in our Epo-TAg^h^ mice it is possible that the K^+^ current was affected by Epo deficiency, which modifies the sensitivity of the RTN. However, there is no data in the literature supporting the possibility that Epo interacts with GPR4 or TASK molecular proton detectors. Further studies are needed to determine how Epo might modulate RTN chemosensitivity. Paracrine stimulation of RTN/pFRG neurons by astrocytes having CO_2_/H^+^-sensing properties has also been implicated in the RTN/pFRG neuron activation under hypercapnia (Gourine et al., 2010; Olivares et al., 2021). Thus, a possible explanation would be that Epo deficiency affects the coupling between astrocytes and neurons in this medullary area. In support of this hypothesis, it has been shown that RTN/pFRG astrocytes responded to physiological decreases in pH with vigorous elevations in intracellular Ca^2+^ (Gourine et al., 2010) and that Epo regulates Ca^2+^ transport in cells (Körbel et al., 2004). It is thus possible that Epo deficiency alters the capacity of RTN/pFRG astrocytes to detect the CO_2_/H^+^-elevation and thus to excite RTN/pFRG neurons. Further experiments are thus necessary to validate this hypothesis. Whatever the cellular explanation, it seems that it is not the CO_2_/H^+^-sensitive cells of the RTN/pFRG which are the origin of a reinforcement of *f*_R_ for moderate levels of hypercapnia. Another explanation could be the decrease in buffering capacity that could be related to the decrease in erythrocytes (Ellison et al., 1958), which would modify the response to CO_2_. Again, further research would be necessary to confirm this hypothesis.

### 4.4 The Hypercapnic Activation of Neurons in the Nucleus of the Solitary Tract, the Main Projection Site From the Peripheral Chemoreceptors, Is Blunted by the Epo Deficiency

In WT mice, hypercapnia elicited an increase in *c-fos* expression in commissural and medial parts of NTS, as already described (Teppema et al., 1997). Thus, the NTS plays a pivotal role as a site of integration of peripheral information, and also as a site of central CO_2_ chemoreception (Dean and Putnam, 2010). The cNTS and mNTS are known to be the major projection site from peripheral chemoreceptors (Torrealba and Claps, 1988; Finley and Katz, 1992), and some cells in the NTS were considered as directly sensitive to CO_2_ [e.g., (Dean et al., 2001; Dean and Putnam, 2010; Fu et al., 2017; Onimaru et al., 2021)]. Therefore, the increase in the number of c-FOS-positive cells under hypercapnia up to its level in WT mice would be associated with the stimulation of peripheral chemoreceptors by CO_2_/H^+^. This is consistent with the reported increase in carotid body sinus nerve firings due to elevation of CO_2_ (Fitzgerald and Dehghani, 1982) and/or to a direct effect of CO_2_/H^+^ stimulus. Therefore, the absence of CO_2_/H^+^-activation in cNTS and mNTS in Epo-TAg^h^ mice suggests that chronic Epo deficiency affects either the functioning of carotid bodies, the integration of inputs from peripheral chemoreceptors or the direct CO_2_/H^+^ NTS cell sensitivity. Whatever the affected pathway, a mutation-dependant dysfunction and/or loss of the involved NTS neurons are possible. This hypothesis is supported both by previous reported interactions between Epo and carotid bodies and the presence of EpoR on cNTS and mNTS cells (Soliz et al., 2005; Andrade et al., 2018). Thus, based on present data it would be interesting in the future to investigate the Epo interaction with carotid bodies in a context of hypercapnia and to explore the properties of NTS neurons because of the influence of Epo/Epo-R signalling in brain development (Yu et al., 2002; Chen et al., 2007).

### 4.5 Epo Deficiency Favours the Hypercapnic Activation of 5-HT and Non 5-HT Neurons of Medullary Raphe Nuclei, a Pathway Possibly Involved in Reinforcement of the HcVR in Epo-TAg^h^ Mice

Epo-TAg^h^ displayed an increase in *c-fos* expression induced by hypercapnia in the medullary raphe nuclei, RPa and RMg, whereas WT mice did not show such an increase. In RPa, but not in RMg, a significant part of the c-FOS-positive cells were also immunoreactive for 5-HT. The activation of 5-HT cells by CO_2_/H^+^ (Figure 3) are in agreement with the idea that medullary 5-HT neurons play a significant role in central chemosensitivity, in particular at the level of *f*_R_ (Iceman et al., 2013; Teran et al., 2014). It is possible that the Epo-deficiency led to a modification of the sensitivity of these 5-HT neurons, thus contributing, at least in part, to an increase in *f*_R_ for a moderate level of CO_2_ not observed in WT mice. In addition, we also found an increase in c-FOS positive cells in medullary raphe nuclei that were not serotoninergic (Figure 3). This observation is consistent with the previously reported non-serotoninergic cells stimulated by CO_2_ in medullary raphe (Iceman and Harris, 2014). These non-5-HT cells are robustly stimulated by CO_2_ and express neurokinin 1 receptors (NK1-R) (Iceman and Harris, 2014) and a lesion of *raphe* NK1-R expressing cells reduces the HcVR (Hodges et al., 2004; Nattie et al., 2004; Nattie and Li, 2006b). It is thus possible that medullary *raphe* 5-HT and non-5-HT/NK1-R expressing cells are involved in the reinforcement of the HcVR in Epo-TAg^h^ mice. These data open up interesting perspectives under clinical situations such as sleep apnea (SA) or central congenital hypoventilation syndrome (CCHS) in which the CO_2_ respiratory adaptation is deficient. SA is a pathological condition characterized by recurrent airway obstruction or cessation of breathing during sleeps resulting in intermittent phases of hypercapnia and hypoxemia (Badran et al., 2014). This sleep disorder is associated with sleep fragmentation, increased blood pressure and heart rate, neurocognitive dysfunction, cardiovascular morbidity, among other things (Young et al., 2002; Gozal, 2013; Badran et al., 2014; Konecny et al., 2014). Finding new therapies based on our findings would be of particular interest. With this in mind, we recently suggested that carbamylated form of human Epo (cEpo) could normalized the cardiorespiratory disorders triggered by intermittent hypoxia which mimic Obstructive SA (Andrade et al., 2021). This result suggested that cEpo could be considered as a new therapy for sleep apneas (Zoccali and Mallamaci, 2021). CCHS is a rare neurorespiratory disease characterized by the absence or a very severe reduction in the CO_2_/H^+^ chemosensitivity leading to profound hypoventilation at least during sleep (Trang et al., 2020). On the basis of data from animal models, the actual consensus is that lack of CO_2_/H^+^ sensitivity is due to a dysfunction or loss of the CO2/H + PHOX2B-positive neurons of the RTN with *a priori* no disturbance at the level of medullary raphe 5-HT and non-5HT neurons (Amiel et al., 2003; Dubreuil et al., 2008; Ramanantsoa et al., 2011). Incidental clinical observations suggested a restoration of CO_2_/H^+^ sensitivity in some CCHS patients who took progestin for contraceptive purposes and data from animal models free from CCHS suggest that progestin stimulates the respiratory drive through an action involving medullary raphe neurons, some of which are 5-HT neurons (Straus et al., 2010; Joubert et al., 2016; Loiseau et al., 2018). Since the present study showed that the modulation of Epo leads to an increase in the sensitivity of 5-HT and non-5-HT neurons of the raphe medullary nuclei leading to stimulation of *f*_R_ for moderate level of CO_2_, it could be interesting to explore if Epo pharmacological manipulation is able to restore a CO_2_/H^+^ respiratory response in preclinical CCHS models.

## 5 Conclusion

We observed that, in adult male mice, Epo-deficiency affects both the pattern of ventilatory response to moderate hypercapnia and the CO_2_/H^+^-activated medullary respiratory network. Our histological data suggest that the exaggeration of the HcVR in Epo-TAg^h^ mice, notably through an effect on *f*_R,_ would imply the recruitment of chemosensitive 5-HT and non 5-HT neurons of the medullary raphe nuclei, which are silent in WT mice. This recruitment seems to be sufficient to counteract a decrease in both the activation of RTN/pFRG neurons and the pathway involving NTS neurons. Of course, other CO_2_/H^+^ chemosensitive areas located in supramedullary regions such as the locus coeruleus and caudal hypothalamus (Dillon and Waldrop, 1993; Horn and Waldrop, 1994, 1994; Nattie, 1999), could also be involved and further investigations are needed to search for their implication. However, with or without the intervention of supramedullary structures, our data suggest that induction of neuroplasticity by changes in the Epo content modulates the ventilatory response to hypercapnia.

## Data Availability

The original contributions presented in the study are included in the article/Supplementary Material, further inquiries can be directed to the corresponding author.

## Abbreviations

7N, facial nucleus; Epo, erythropoietin; EpoR, erythropoietin receptor; fR, respiratory frequency; Hc, hypercapnia; HcVR, hypercapnic ventilatory response; NK1-R, neurokinin-1 receptor; NOS, nitric oxide systems; cNTS, commissural part of the nucleus of the solitary tract; mNTS, medial part of the nucleus of the solitary tract; vlNTS, ventrolateral part of the nucleus of the solitary tract; PHOX2B, paired-like homeobox 2b; RMg, raphe magnus nucleus; ROb, raphe obscurus nucleus; RPa, raphe pallidus nucleus; RTN/pFRG, retrotrapezoid nucleus/parafacial respiratory group; V̇E, minute ventilation; cVLM, caudal ventrolateral reticular nucleus of the medulla; rVLM, rostral ventrolateral reticular nucleus of the medulla; VMS, ventral medullary surface; VT, tidal volume.
